# Effects of a Mobile Health Intervention Based on Behavioral Integrated Model on Cognitive and Behavioral Changes in Gestational Weight Management: Randomized Controlled Trial

**DOI:** 10.2196/55844

**Published:** 2025-03-10

**Authors:** Meng Zhou, Li Wang, Ying Deng, Jinjin Ge, Shiqi Zhao, Hua You

**Affiliations:** 1 Department of Humanities and Management School of Nursing Nanjing Medical University Nanjing China; 2 School of Nursing Anhui Medical University Hefei China; 3 Department of Gynaecology and Οbstetrics Changzhou Maternal and Child Health Care Hospital Changzhou China; 4 Department of Social Medicine and Health Education School of Public Health Nanjing Medical University Nanjing China

**Keywords:** cognition, health behavior, information-motivation-behavioral skills model, mobile health, psychological models, pregnant woman, randomized controlled trial, mobile phone

## Abstract

**Background:**

The key to gestational weight management intervention involves health-related behaviors, including dietary and exercise management. Behavioral theory-based interventions are effective in improving health-related behaviors. However, evidence for mobile health interventions based on specific behavioral theories is insufficient and their effects have not been fully elucidated.

**Objective:**

This study aimed to examine the effects of a gestational mobile health intervention on psychological cognition and behavior for gestational weight management, using an integrated behavioral model as the theoretical framework.

**Methods:**

This study was conducted in a tertiary maternity hospital and conducted as a single-blind randomized controlled trial (RCT) in Changzhou, Jiangsu Province, China. Using the behavioral model, integrated with the protection motivation theory and information–motivation–behavioral skills model (PMT-IMB model), the intervention group received a mobile health intervention using a self-developed app from 14 to 37 gestational weeks, whereas the control group received routine guidance through the application. Psychological cognition and behaviors related to weight management during pregnancy were the main outcomes, which were measured at baseline, and at the second and third trimesters of pregnancy using a self-designed questionnaire. Generalized estimation and regression equations were used to compare the outcome differences between the intervention and control groups.

**Results:**

In total, 302 (302/360, 83.9%) participants underwent all measurements at 3 time points (intervention group: n=150; control group: n=152). Compared with the control group, the intervention group had significantly higher scores for information, perceived vulnerability, response cost, and exercise management in the second trimester, while their scores for perceived vulnerability, response cost, and diet management were significantly higher in the third trimester. The results of repeated measures analysis revealed that, in psychological cognition, the information dimension exhibited both the time effects (T3 β=3.235, 95% CI 2.859-3.611; *P*<.001) and the group effects (β=0.597, 95% CI 0.035-1.158; *P*=.04). Similarly, response costs demonstrated both the time effects (T3 β=0.745, 95% CI 0.199-1.291; *P*=.008) and the group effects (β=1.034, 95% CI 0.367-1.700; *P*=.002). In contrast, perceived vulnerability solely exhibited the group effects (β=0.669, 95% CI 0.050-1.288; *P*=.03). Regarding weight management behaviors, both time (T3 β=6, 95% CI 4.527-7.473; *P*<.001) and group (β=2.685, 95% CI 0.323-5.047; *P*=.03) had statistically significant impacts on the total points. Furthermore, the exercise management dimension also demonstrated both the time effects (T3 β=3.791, 95% CI 2.999-4.584; *P*<.001) and the group effects (β=1.501, 95% CI 0.232-2.771; *P*=.02).

**Conclusions:**

The intervention program was effective in increasing psychological cognitions in terms of information, perceived vulnerability, and response costs, as well as promoting healthy behaviors among Chinese pregnant women. This study provides new evidence supporting the effectiveness of mobile intervention based on behavioral science theory in gestational weight management.

**Trial Registration:**

Chinese Clinical Trial Registry ChiCTR2100043231; https://www.chictr.org.cn/showproj.html?proj=121736

## Introduction

Inappropriate gestational weight gain (GWG) remains a public health problem requiring immediate attention [1,2]. The proportion of inadequate weight gain among pregnant women is approximately 20% in the United States, Canada, and Europe, whereas the proportion of excessive weight gain is approximately 50%. Moreover, in Asia, 31% of pregnant women experience inadequate weight gain, whereas 37% experience excessive weight gain [3]. Currently, the appropriate GWG rate in China is less than 50% [4,5]. Appropriate GWG is essential, as both excessive and inadequate weight gain pose adverse risks to maternal and child health [6-9].

Mobile health refers to the use of mobile technologies including mobile phones, personal digital assistants, and even tablet computers to improve patient health [10]. In the current era of rapidly advancing information technology, mobile health is widely used in various medical fields. Despite the identification of various challenges in mobile health during its extensive application, including the lack of regulatory oversight, limited evidence-based literature, and concerns about privacy and security [11], mobile health still has many advantages. It not only overcomes the limitations of time and space but also saves medical and human resources. Notably, mobile health technology has become a popular resource for pregnant women to learn dietary and lifestyle modifications [12]. Mobile health interventions based on gestational diet and physical activity are widely used to promote weight management behavior [13,14], and several reviews have summarized the effectiveness of this emerging internet intervention approach to promote maternal and infant health [10,15,16].

Unhealthy behaviors, such as overeating, physical inactivity, and a sedentary lifestyle during pregnancy, are known to be considerably associated with inappropriate GWG [17]. The key to pregnancy weight management intervention is the promotion of health-related behaviors. Interventions based on health behavioral theory are effective in controlling GWG. For instance, the social cognitive theory [18,19], health belief model [20], and integrated theory of health behavioral change [21], have been used to formulate mobile health interventions aimed at gestational weight management. However, the effects of interventions based on behavioral theory are not universal. For example, the behavioral lifestyle intervention based on social cognitive theory had no impact on gestational weight management in African American individuals with obesity and White individuals [22]. Hence, the effects of these gestational weight management interventions based on behavioral theory have not been fully elucidated; therefore, in the context of mobile interventions targeting GWG, it is also necessary to further explore and validate potentially valuable behavioral theories in combination with practical applications.

**Figure 1:**
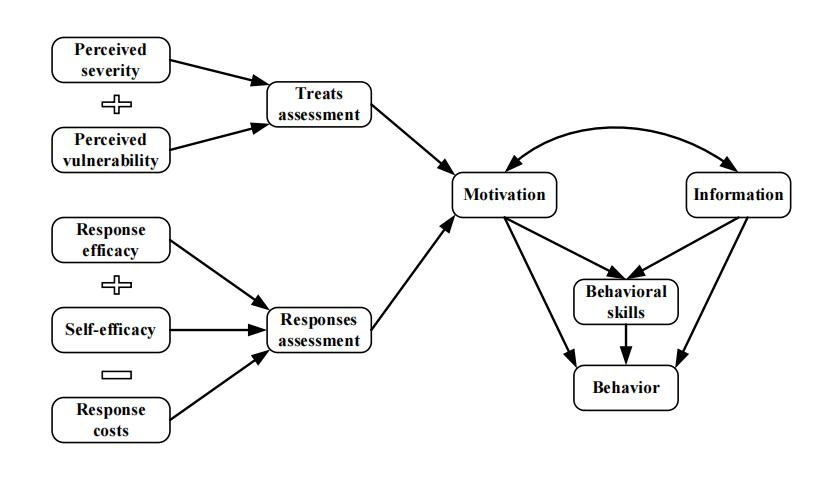
The behavior model integrated by protection motivation theory and information–motivation– behavioral skills model.

In this study, the behavioral model integrated with the protection motivation theory and information–motivation–behavioral skills model (PMT-IBM model) was used to guide the mobile health intervention for gestational weight management. The integrated model involves 8 dimensions: information, behavioral skill, motivation, perceived severity, perceived vulnerability, response efficacy, self-efficacy, and response cost (Figure 1). In this integrated model, the 5 dimensions that explain the generation of motivation in the original protective motivation theory (PMT), namely perceived severity, perceived vulnerability, response efficacy, self-efficacy, and response cost, replace the single motivation dimension in the information-motivation-behavioral skills (IMB) model. These dimensions interact with the information dimension and then either directly influence behavior change or indirectly impact the emergence of behavior through behavioral skill factors [23]. Both PMT and IMB revolve around motivation. Specifically, PMT focuses on the motivation or willingness behind behavior, elucidating the formation of preventive behavioral motivation from a psychological perspective [24]. The IMB model further integrates motivation with information and skills [25]. While gestational weight control is a preventive and beneficial behavior adopted by individuals to safeguard their health [26]. Whether or not individuals have the motivation to engage in behaviors such as maintaining a balanced diet and engaging in moderate exercise during pregnancy reflects their subjective willingness to adopt such protective measures [14]. Therefore, this integrated model is suitable for the development of health education programs for gestational weight control among pregnant women. In a previous independent study, we have already extensively validated the integrated model’s fitness and ability to interpret gestational weight management behavior in a cross-sectional survey of 525 pregnant women. The study explains the influencing mechanisms underlying gestational weight management behavior at the individual psychological level [27]. Nevertheless, this initially proven interpretive integrated model has not yet been verified for its effectiveness in guiding interventions in practice.

Therefore, this trial aimed to determine whether a mobile health intervention applying the PMT-IBM model as the framework could enhance psychological cognitions and behaviors toward gestational weight management.

## Methods

### Study Design

This was a single-blind randomized controlled trial (RCT). Eligible participants were randomly assigned by computer to (1) intervention (mobile health based on the integrated model) and (2) control (routine guidance through the mobile application) groups. The study was prospectively registered with the Chinese Clinical Trial Registry (ChiCTR2100043231).

### Study Setting and Participants

Between August and December 2021, participants were recruited from Changzhou, one of the central cities in China’s Yangtze River Delta region and a developed city in southern Jiangsu Province. The city occupies an area of approximately 4372.15 km^2^, with a per capita gross domestic product of more than US $25,000 in 2021. (Changzhou Municipal People’s Government, 2022) The study site was a tertiary maternity hospital where approximately 40% of the deliveries in this city are performed (Changzhou Maternal and Child Health Care Hospital, 2021). This study used purposive sampling, where the researchers interning in the obstetrics and gynecology outpatient clinic invited all eligible pregnant women to participate in the study. The inclusion and exclusion criteria are listed in Textbox 1.

**Inclusion and exclusion criteria.**

**Inclusion criteria**

- singleton pregnancy;
- pregnancy confirmed by a pregnancy test (gestational age ≤14 weeks);
- intention to live in the region until delivery;
- ability to complete the questionnaire; and
- agreement to participate and provide written informed consent.

**Exclusion criteria**

- age <18 years;
- history of neurological, cardiovascular, hepatic, and renal medical complications;
- essential hypertension and diabetes mellitus; and
- other complications, such as deafness and dumbness.

### Sample Size

Distinct from the protocol, there has been a change in the outcome indicator used for calculating the sample size in this study. Initially, in the protocol, both GWG and weight management behaviors were primary outcome indicators, and the outcome indicator for sample size calculation was based on data related to GWG in Jiangsu Province. However, due to the pandemic, the final sample size did not meet the requirements for analyzing weight outcomes. Since this study primarily focuses on behavioral changes among pregnant women, the sample size was calculated based on the behavioral indicator (the score on the Gestational Weight Management Behavior Scale). The score, serving as both a behavioral indicator and another primary outcome variable in the protocol, is deemed suitable for sample size calculation in this analysis. The formula for calculating sample size was based on a 2-sample mean comparison: n1=n2=2[ (Z_α_+ Z_β_)s/d]^2^ [28]. The presurvey results were used to obtain the s=11.8 and d=5.25 [27]. Therefore, the estimated sample size was as follows: n1=n2=108. A 20% attrition rate was considered for this study; therefore, the required sample size was increased to 270 participants, including 135 participants in each group.

### Randomization and Blinding

In this single-blind RCT, the participants were blinded. The randomization sequence was generated using SPSS software (version 25.0; IBM Corp) by a research assistant who did not participate in the recruitment process. Random numbers were matched to the intervention app. When participants completed the baseline questionnaire, they were assigned a unique registration number. Thereafter, the backend of the app consolidated the participants’ information to screen those who met the inclusion criteria. The registration number was then matched against the groups’ random sequence, to categorize the participants into intervention or control groups.

### Data Collection

Our research team comprehensively studied the operational definitions of each dimension of the PMT-IBM model, as well as designed the questionnaires to strictly comply with the questionnaire design process [27]. Three trained outcome assessors performed face-to-face baseline data collection during the first trimester (T1: gestational age of ≤14 weeks) at the maternity clinic. The follow-up data were collected using a questionnaire link provided on the mobile health app in the second trimester (T2: gestational age of 27-28 weeks) and third trimester (T3: gestational age of ≥37 weeks). During questionnaire completion, the assessors reminded the participants to self-report their actual preceding month circumstances.

### Outcome Measures

A detailed baseline evaluation was performed for all participants before initiating the study. The evaluation included the following three parameters: (1) main demographic characteristics: including maternal age, education level, parity, height, and prepregnancy weight. (2) Gestational psychological cognition scales: included 7 dimensions (information, behavioral skills, perceived severity, perceived vulnerability, response efficacy, self-efficacy, and response costs), which were designed by the PMT-IMB model. Each dimension was measured on a 5-point Likert scale (from 1=absolutely disagree to 5=absolutely agree) and included 5 items each, with a total score of 5-25. A higher total score indicated better psychological cognition among pregnant women. The total Cronbach α for the cognition scale of 0.860, ranged from 0.822 to 0.938. (3) Gestational weight management behavioral scales included 4 dimensions encompassing 20 items, each item was rated on a 5-point Likert scale ranging from 1 (never) to 5 (always). Specifically, exercise management dimension (consisting of 9 items, with a score range of 9-45) pertains to diverse exercise management behaviors adopted by pregnant women to maintain optimal GWG, encompassing the duration and type of exercise. The dietary management dimension (including 4 items, with a score range of 4-20) refers to a series of dietary management practices implemented by pregnant women to achieve appropriate GWG. The self-monitoring and regulation dimension (comprising 4 items, with a score range of 4-20) entails pregnant women engaging in weight monitoring, diet tracking, self-regulation, and other related behaviors aimed at maintaining suitable GWG. Then, management objectives (containing 3 items, with a score range of 3-15) involve pregnant women setting their own weight gain, dietary, and exercise goals in accordance with appropriate standards for GWG. Scores were analyzed using the gross for each dimension, with higher scores indicating superior behavioral management in each respective dimension. The total Cronbach α for the behavioral scale of 0.844, ranged from 0.653 to 0.866. The validity test of the scale has been reported in the previous explanatory study [27]. The detailed information for the scales is shown in Multimedia Appendix 1 and the operational definition of gestational weight management behaviors is shown in Multimedia Appendix 2.

### Intervention Tool

The mobile health intervention was implemented using a self-developed app, “Pregnancy Assistant.” “Pregnancy Assistant” was collaboratively developed by project team members, obstetricians, and computer engineers. The project leader provided the application development framework and relevant requirements, while the obstetricians were responsible for gathering feedback and suggestions from clinical health care professionals regarding the development of a “Pregnancy Assistant.” The project members, on the other hand, were tasked with collecting information on pregnancy weight management and understanding the weight management needs of pregnant women.

The application comprises 3 primary user end points: for pregnant women, hospitals, and the integrated system database. The client for pregnant women included modules such as gestational age, a questionnaire, health education, notification, and weight records. Among these, the gestational age, weight records, and questionnaire modules serve as intervention assistance tools. The most crucial intervention involves delivering health education content through the “health education” module, tailored to the dimensions of the model and the specific gestational weeks of the pregnant women, along with corresponding notification reminders. In the notification module, pregnant women can engage in communication and share their insights and experiences through the created groups. The hospital client could upload health education-related materials. All survey data were downloaded and stored in the database. In the health education module, the content received by the intervention group differs from that of the control group. Specifically, the control group is sent to routine pregnancy health care guidance, whereas the intervention group receives materials that are designed according to the PMT-IMB model (Multimedia Appendix 3).

### Control Group

The control group underwent routine prenatal care in accordance with the guidelines for preconception care and prenatal care in China provided by the hospital [29]. This prenatal care encompassed regular prenatal examinations, periodic antenatal information services, and health education and guidance. Notably, the health education component only incorporated information that the country mandates to be imparted to pregnant women. Some of these health education information services would be delivered through the app named Pregnancy Assistant to ensure convenient access to and timely updates of the information.

### Intervention Group

In addition to implementing the same routine prenatal care as those provided to the control group, the intervention group implemented specific interventions tailored to the dimensions and corresponding definitions of the behavioral model. These interventions were delivered through a variety of formats, including image texts, videos, expert lectures, and peer communication. According to the research results on the model construction [27], the main contents of the interventions were determined based on the information, motivation, and behavioral skills dimensions. First, we developed various health intervention materials under the 7 psychological cognition dimensions by referring to studies on gestational weight management [29] and gestational guidelines on nutrition [30-32] and exercise [30,33], combined with the recommendations from clinical nutrition experts, obstetricians, and nurses. The intervention materials (ie, image, text, and video) were delivered through the “Pregnancy Assistant” app, (24 items in total, once a week) during the intervention period, which spanned from the 14th to the 37th week of gestation. Throughout this process, researchers also regularly reminded pregnant women via the notification module of the app and WeChat (Tencent) to use the intervention materials to improve compliance. Second, every 2 weeks, a 45-minute expert lecture was held in the application to introduce information regarding the risks and severity of inappropriate GWG, its epidemiological knowledge such as risk factors, susceptible populations, and suitable preventive dietary and exercise methods. Following each expert lecture, a 15-minute question-and-answer session was offered to participants to address existing obstacles against weight management. In addition, to boost confidence and stimulate healthy behavioral changes, experienced participants were encouraged to share their thoughts and experiences via the communication group. The detailed intervention content is shown in Multimedia Appendix 3. The study adheres to the CONSORT-EHEALTH (Consolidated Standards of Reporting Trials of Electronic and Mobile Health Applications and Online Telehealth) guidelines, and the completed CONSORT-EHEALTH checklist is available in the Multimedia Appendix 4.

### Statistical Analysis

In the descriptive statistical analysis, continuous variables are expressed as mean values and (SDs). Use the Kolmogorov-Smirnov test to verify the normality of these data. For data that met normality, a 2-sample independent *t* test was used to compare the 2 groups, while (ANOVA was used to compare multiple groups. For data that do not meet normality, nonparametric tests were used. Categorical variables, presented as frequencies (%), are compared between groups using the Pearson chi-square test. In a general linear model, the cognition dimensions in the PMT-IMB model and weight management behavioral dimensions were considered as the dependent variables. Intervention or not was considered the primary independent variable (0 for the control group and 1 for the intervention group). Covariates included age, education level, parity, and prepregnancy BMI. Each participant completed the same questionnaire thrice, providing repeatable and dependable measurement data. Therefore, the differential changes in psychological cognitions and behaviors at T2 and T3 with respect to T1 between the 2 groups were assessed using generalized estimating equations. When no statistical interaction between time and group was present, we only included the group’s main effect and time terms to demonstrate the intervention’s effect. Those who had a miscarriage or premature birth during follow-up were excluded from the final data analysis. Therefore, intention-to-treat analysis was not used in the analysis of this study. All data analyses were performed using IBM SPSS Statistics for Windows (version 25.0), and statistical significance was set at *P*<.05 (2-sided).

### Ethical Considerations

This study was approved by the Ethics Committee of Nanjing Medical University (number NMU 2020-63). All participants were comprehensively informed of the voluntary nature of their participation and their right to withdraw from the study at any time. The investigation was performed only after written informed consent had been obtained from the participants. We adhere strictly to confidentiality regarding the obtained information, and we have committed to respecting patients’ privacy and confidentiality rights during the data collection process. As a gesture of appreciation for the time spent by the participants completing the questionnaire, US $8.4 was provided to each participant.

## Results

### Participant Recruitment and Retention

**Figure 2:**
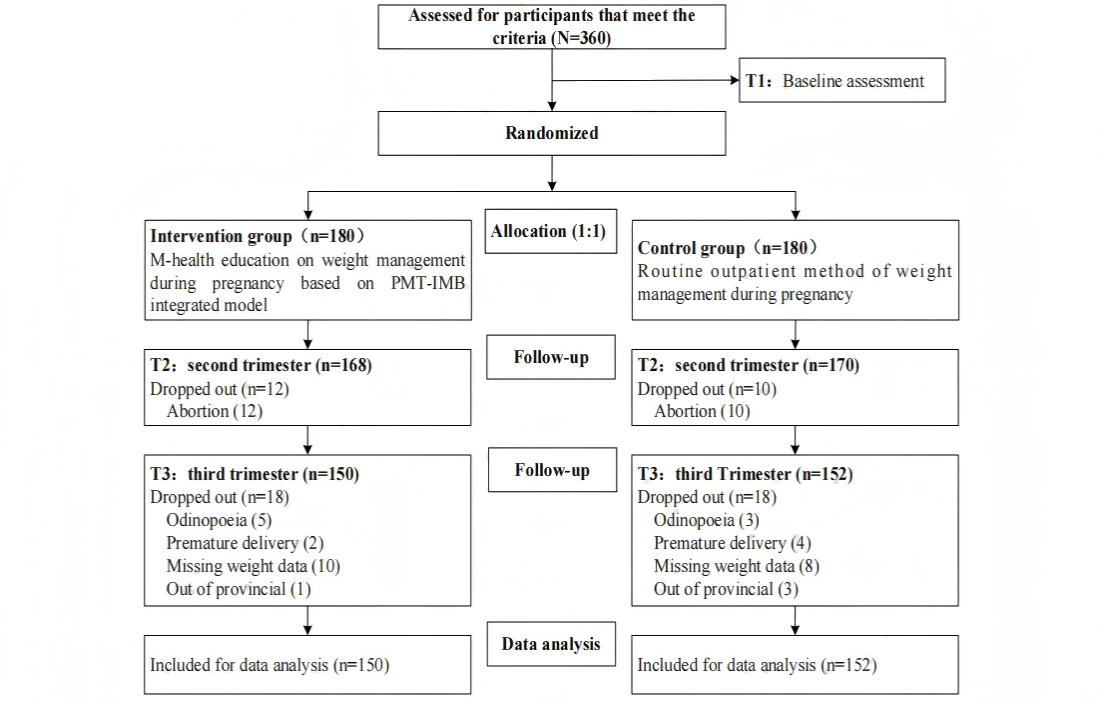
Flow chart of the assessment, allocation, reasons for withdrawal, and number of participants included in the data analysis.

After evaluation, 360 participants satisfied the eligibility criteria at baseline and were randomly assigned to intervention and control groups (n=180 per group). After their inclusion in the study, pregnant women registered themselves on the app using their mobile phones and completed the baseline questionnaire. The T2 follow-up involved 338 participants (intervention group: n=168; control group: n=170; retention rate: 88.9%). By T3, 302 participants with complete follow-up data were included in the final data analysis (intervention group: n=150; control group: n=152). The overall retention rate was 83.9%. Participants who dropped out at each stage of the study are shown in Figure 2. The comparison of demographic and clinical characteristics between dropouts and completers of the intervention revealed no significant differences (*P*>.05 for all comparisons).

### Main Demographic Characteristics

The participants’ demographic characteristics are presented in Table 1. Among the 302 participants, the mean ages of the intervention and control groups were 29.31 (SD 3.38) and 29.16 (SD 3.49) years, respectively. In both groups, more than half of the participants had at least a college education (59.3%, 89/150 vs 54.6%, 83/152; *P*=.41). Most participants were primipara, and the proportion of participants with an appropriate prepregnancy BMI also exceeded 70%. No statistically significant between-group differences were observed in the main demographic characteristics (*P*>.05).

**Table 1:** Main demographic characteristics between the intervention group and control group (N=302).

Characteristics			Intervention group (N=150)		Control group (N=152)		Z			Chi-square (*df*)			*P* value	
Age (years), mean (SD)			29.31 (3.38)		29.16 (3.49)		–0.116			—^a^			.91	
**Education level^b^, n (%)**								—			0.688 (1)			.41
	College education and above	89 (59.3)		83 (54.6)										
	Junior college and below	61 (40.7)		69 (45.4)										
**Parity, n (%)**								—			0.040 (1)			.84
	Multiparity	39 (26.0)		38 (25.0)										
	Primiparity	111 (74.0)		114 (75.0)										
Pre-pregnancy BMI (kg/m^2^), mean (SD)			21.16 (2.62)		21.25 (2.57)		–0.324			—			.75	
**Prepregnancy BMI Classification, n (%)**								—			0.744 (3)			.87
	underweight	19 (12.7)		17 (11.2)										
	normal weight	110 (73.3)		116 (76.3)										
	overweight	18 (12.0)		15 (9.9)										
	obese	3 (2.0)		4 (2.6)										

^a^Not applicable.

^b^Junior college degree is the median level of education.

### Intervention Effect on Psychological Cognitions and Weight Management Behaviors

As shown in Table 2, at baseline, only the management objective dimension (adjusted β=0.686, *P*=.04) was significant, whereas all dimensions of psychological cognition and weight management behavior were not significantly different between the 2 groups. Adjusted general linear models demonstrated that participants in the intervention group had noticeably higher scores for information (adjusted β=0.836, *P*=.02), perceived vulnerability (adjusted β=0.894, *P*=.03), response cost (adjusted β=1.101, *P*=.006), and exercise management (adjusted β=2.089, *P*=.01) at T2 than their counterparts in the control group. Furthermore, their scores for perceived vulnerability (adjusted β=1.078, *P*=.01), response cost (adjusted β=1.538, *P*=.001), and dietary management (adjusted β=0.959, *P*=.007) were statistically significantly higher at T3. Although behavioral skills, perceived severity, response efficacy, self-efficacy, self-monitoring and regulation, and management objectives yielded generally better scores in the intervention group than in the control group at T2 and T3, statistical significance was not achieved.

**Table 2:** The intervention effect of psychological cognition and weight management behaviors is based on adjusted general linear models (N=302).

Outcomes				Intervention group (N=150)		Control group (N=152)		Adjusted β^a^ (95% CI)		*P* value
				Mean (SD)		Mean (SD)				
**Psychological cognitions**										
	**Information**									
		T1^b^	13.77 (3.10)		13.55 (2.90)		0.174 (–0.500 to 0.849)		.61	
		T2^c^	16.54 (2.96)		15.69 (3.23)		0.836 (0.139 to 1.534)		.02	
		T3^d^	17.25 (3.02)		16.53 (3.28)		0.678 (–0.031 to 1.387)		.06	
	**Behavioral skills**									
		T1	17.11 (3.76)		16.36 (3.97)		0.731 (–0.144 to 1.606)		.10	
		T2	17.30 (3.61)		16.71 (3.93)		0.615 (–0.234 to 1.465)		.16	
		T3	18.31 (4.14)		17.81 (4.04)		0.483 (–0.434 to 1.401)		.30	
	**Perceived severity**									
		T1	21.24 (4.60)		21.36 (4.45)		–0.175 (–1.184 to 0.834)		.73	
		T2	21.95 (3.82)		21.90 (3.60)		0.035 (–0.807 to 0.877)		.93	
		T3	21.72 (3.83)		21.55 (3.61)		0.109 (–0.722 to 0.939)		.80	
	**Perceived vulnerability**									
		T1	19.32 (3.50)		19.31 (3.63)		0.009 (–0.803 to 0.821)		.98	
		T2	19.54 (3.23)		18.63 (3.65)		0.894 (0.113 to 1.675)		.03	
		T3	19.66 (3.56)		18.58 (3.89)		1.078 (0.231 to 1.924)		.01	
	**Response efficacy**									
		T1	21.81 (3.68)		22.28 (3.72)		–0.523 (–1.351 to 0.305)		.22	
		T2	21.93 (3.54)		21.61 (4.09)		0.313 (–0.553 to 1.178)		.48	
		T3	21.85 (3.65)		21.47 (3.77)		0.347 (–0.493 to 1.187)		.42	
	**Self-efficacy**									
		T1	19.94 (3.88)		20.14 (3.94)		–0.233 (–1.120 to 0.655)		.61	
		T2	18.89 (3.94)		18.64 (4.08)		0.326 (–0.575 to 1.227)		.48	
		T3	19.01 (4.57)		18.7 (4.18)		0.342 (–0.649 to 1.334)		.50	
	**Response cost**									
		T1	17.13 (4.09)		16.65 (4.55)		0.492 (–0.483 to 1.467)		.32	
		T2	18.15 (3.33)		17.05 (3.51)		1.101 (0.325 to 1.876)		.006	
		T3	18.40 (3.58)		16.88 (4.13)		1.538 (0.663 to 2.414)		.001	
**Weight management behaviors**										
	**Exercise management**									
		T1	22.81 (5.71)		21.87 (6.22)		0.849 (–1.489 to 2.187)		.21	
		T2	25.66 (6.75)		23.55 (7.50)		2.089 (–0.477 to 3.702)		.01	
		T3	26.86 (7.47)		25.41 (7.13)		1.420 (–0.238 to 3.078)		.09	
	**Diet management**									
		T1	13.33 (3.59)		13.1 (3.32)		0.166 (–0.602 to 0.935)		.67	
		T2	12.67 (3.42)		12.59 (3.18)		–0.003 (–0.729 to 0.724)		.99	
		T3	13.68 (2.79)		12.64 (3.41)		0.959 (0.269 to 1.648)		.007	
	**Self-monitoring and regulation**									
		T1	10.91 (3.44)		10.60 (3.52)		0.235 (–0.540 to 1.009)		.55	
		T2	11.08 (3.26)		10.89 (3.2)		0.119 (–0.588 to 0.826)		.74	
		T3	12.31 (3.32)		11.87 (3.21)		0.394 (–0.333 to 1.120)		.29	
	**Management objectives**									
		T1	6.65 (2.97)		5.94 (2.86)		0.686 (0.024 to 1.349)		.04	
		T2	6.40 (2.89)		6.14 (2.82)		0.229 (–0.416 to 0.875)		.49	
		T3	7.37 (3.30)		7.07 (2.77)		0.272 (–0.415 to 0.958)		.44	
	**Total scores**									
		T1	53.70 (11.07)		51.51 (11.52)		1.936 (–0.571 to 4.444)		.13	
		T2	55.81 (12.32)		53.18 (12.83)		2.435 (–0.368 to 5.239)		.09	
		T3	60.22 (13.57)		56.99 (13.46)		3.044 (0.006 to 6.081)		.05	

^a^Model was adjusted for covariables, including age, education level, parity, and prepregnancy BMI. The reference group comprised the control group.

^b^T1: first trimester.

^c^T2: second trimester.

^d^T3: third trimester.

### Generalized Estimating Equations for Psychological Cognition and Weight Management Behaviors

Psychological cognition and weight management behaviors were used as dependent variables, while time and group were used as independent variables in the generalized estimating equation analysis (Table 3)**.** Regarding psychological cognitions, a statistically significant group effect on information (β=0.597, 95% CI 0.035-1.158; *P*=.04), perceived vulnerability (β=0.669, 95% CI 0.050-1.288; *P*=.03), and response cost (β=1.034, 95% CI 0.367-1.700; *P*=.002) were observed. Moreover, time had a statistically significant effect on information (T2: β=2.457, 95% CI 2.086-2.832; *P*<.001; T3: β=3.235, 95% CI 2.859-3.611; *P*<.001) and response cost (T2: β=0.702, 95% CI 0.203-1.201; *P*=.01; T3: β=0.745, 95% CI 0.199-1.291; *P*=.01). However, no significant differences were noted in the perceived severity and response efficacy dimensions among the groups and times.

**Table 3:** Changes in psychological cognition and weight management behaviors in the second trimester (T2) and third trimester (T3) in the control and intervention group compared to baseline first trimester (T1) based on generalized estimating equation models.

Outcomes and independent variables^a^			β (95% CI)	*P* value
**Information**				
	Group (category: intervention)		0.597 (0.035 to 1.158)	.04
	**Time**			
		T2^b^	2.457 (2.086 to 2.832)	＜.001
		T3^c^	3.235 (2.859 to 3.611)	＜.001
**Behavioral skills**				
	Group (category: intervention)		0.613 (–0.091 to 1.317)	.09
	**Time**			
		T2	0.268 (–0.179 to 0.715)	.24
		T3	1.321 (0.818 to 1.824)	＜.001
**Perceived severity**				
	Group (category: intervention)		0.032 (–0.686 to 0.751)	.93
	**Time**			
		T2	0.632 (0.121 to 1.124)	.02
		T3	0.331 (–0.181 to 0.843)	.21
**Perceived vulnerability**				
	Group (category: intervention)		0.669 (0.050 to 1.288)	.03
	**Time**			
		T2	–0.235 (–0.675 to 0.204)	.29
		T3	–0.199 (–0.690 to 0.293)	.43
**Response efficacy**				
	Group (category: intervention)		0.082 (–0.602 to 0.765)	.82
	**Time**			
		T2	–0.275 (–0.726 to 0.176)	.23
		T3	–0.387 (–0.803 to 0.029)	.07
**Self-efficacy**				
	Group (category: intervention)		0.120 (–0.607 to 0.847)	.75
	**Time**			
		T2	–1.275 (–1.798 to –0.751)	＜.001
		T3	–1.189 (–1.687 to –0.690)	＜.001
**Response cost**				
	Group (category: intervention)		1.034 (0.367 to 1.700)	.002
	**Time**			
		T2	0.702 (0.203 to 1.201)	.006
		T3	0.745 (0.199 to 1.291)	.008
**Exercise management**				
	Group (category: intervention)		1.501 (0.232 to 2.771)	.02
	**Time**			
		T2	2.262 (1.489 to 3.034)	＜.001
		T3	3.791 (2.999 to 4.584)	＜.001
**Diet management**				
	Group (category: intervention)		0.448 (–0.121 to 1.017)	.12
	**Time**			
		T2	–0.579 (–1.003 to –0.156)	.007
		T3	–0.053 (–0.506 to 0.400)	.82
**Self-monitoring and regulation**				
	Group (category: intervention)		0.313 (–0.275 to 0.900)	.30
	**Time**			
		T2	0.235 (–0.192 to 0.663)	.28
		T3	1.338 (0.891 to 1.784)	＜.001
**Management objectives**				
	Group (category: intervention)		0.423 (–0.091 to 0.937)	.11
	**Time**			
		T2	–0.026 (–0.415 to 0.362)	.89
		T3	0.924 (0.546 to 1.302)	＜.001
**Total points**				
	Group (category: intervention)		2.685 (0.323 to 5.047)	.03
	**Time**			
		T2	1.891 (0.524 to 3.258)	.007
		T3	6.000 (4.527 to 7.473)	＜.001

^a^For the group, the control group was the reference; for time, the first trimester (T1) was the reference.

^b^T2: second trimester.

^c^T3: third trimester.

Regarding weight management behaviors, the 2 groups had a statistically significant effect on exercise management (β=1.501, 95% CI 0.232-2.771; *P*=.02). Furthermore, time had a statistically significant effect on exercise management (T2: β=2.262, 95% CI 1.489-3.034; *P*<.001; T3: β=3.791, 95% CI 2.999-4.584; *P*<.001) and dietary management (T2: β=–0.579, 95% CI 0.818-1.824; *P*=.01). Regarding total weight management behavioral scores, the group (β=2.685, 95% CI 0.323-5.047; *P*=.03) and time (T2: β=1.891, 95% CI 0.524-3.258; *P*=.01; T3: β=6.000, 95% CI 4.527-7.473; *P*<.001) effects were all statistically significant.

## Discussion

### Principal Findings

To the best of our knowledge, this RCT is the first to use mobile health intervention guided by the novel PMT-IBM behavioral theory and test its effectiveness on gestational weight management. Although the mechanism underlying this phenomenon requires further research, statistically significant results suggest that mobile intervention potentially plays positive roles in raising psychological cognitions in weight management including information, perceived vulnerability, and response cost, as well as promoting dietary and exercise management during pregnancy.

### Role of Mobile Health App

In this study, the mobile health app “Pregnancy Assistant” played a crucial role in intervening with the weight management of pregnant women. First, the app served as an effective medium for disseminating information. It provided different health education information to both the control and intervention groups. In addition to the routine pregnancy care information available to both groups, the information provided to the intervention group was designed based on behavioral models, making it more targeted and systematic [34]. This difference might have led to varying intervention effects between the 2 groups. The precision of health education information in the intervention group made it easier for pregnant women to clearly understand the weight control goals and methods at different stages of pregnancy, whereas the control group lacked such precision [35]. Second, the app met the social interactive needs of pregnant women. The regular health lectures attended by the intervention group broadened their knowledge base. The question-and-answer sessions following the lectures addressed participants’ concerns, offering personalized guidance that enhanced their confidence and ability in weight management [36]. The peer support groups within the notification module created a supportive social environment. Pregnant women shared experiences and encouraged each other through communication, and this model of coexisting peer pressure and support could improve their enthusiasm and compliance in weight management [37]. Of course, the convenience of the app should not be overlooked. Pregnant women could access the app anytime, anywhere, via smartphones to obtain necessary information and participate in interactions [38]. This convenience integrated weight management into pregnant women’s daily lives, breaking time and space constraints and helping to form long-term, stable health management habits.

### Effect on Psychological Cognitions

Based on our findings, promoting healthy behaviors in terms of information, perceived vulnerability, and response cost, via mobile health, are of practical relevance. Mobile health technologies such as apps can be applied to the design of the content of mobile interventions for these important psychological cognition dimensions. The intervention content should focus on increasing awareness regarding GWG and control and perceived vulnerability to adverse outcomes and risks while decreasing perceptions of difficult decision-making and personal insecurities. However, no statistically significant between-group differences were noted in the behavioral skills, perceived severity, self-efficacy, and response efficacy dimensions. This may be because most pregnant women lack awareness and hold misguided beliefs regarding weight gain and management. Moreover, a general lack of awareness regarding the specific risks of being overweight or obese during pregnancy is also an important barrier to appropriate GWG [39]. Meanwhile, most pregnant women lack persistence in long-term dietary management and physical exercise and have limited ability to control weight and achieve exercise compliance based on healthy behaviors in reality [40,41]. Furthermore, some pregnant women insufficiently recognize the seriousness of inappropriate GWG, mistakenly believing that pregnancy is perilous by nature and that the occurrence of complications is random [42]. In addition, due to the free availability of extensive information on the internet, the control group also had extra opportunities to access health information or learn it on their own from other sources, which may have affected the accurate evaluation of the intervention. The effectiveness of interventions informed by the theory may be affected by how successfully the theory is applied to the mobile intervention strategies. Although theories are ideal for explaining and validating behavior, they may not be excellent for guiding mobile interventions because of several complicated variables, including time of exposure and environment (ie, exposure to advertisements promoting fast food and sugary beverages). Therefore, this may result in weak or limited effects on cognitive and behavioral changes [43]. Furthermore, in the process of developing the mobile intervention, attention should be paid to the environment and intensity of intervention; moreover, differences in social, economic, cultural, and other factors should be considered [44].

### Effect on Gestational Exercise and Dietary Behaviors

Our findings indicate that the intervention group’s exercise management significantly improved at T2. Evidence from a previous meta-analysis also suggests that prenatal exercise may be an effective method to promote appropriate birth weight of newborns and GWG [45]. In this study, exercise behavior was measured only by the gestational weight management behavioral scales, and future studies should include objective measures of physical activity (ie, step number or physical activity measuring tools) at different stages of pregnancy to evaluate the potential of mobile health intervention in improving gestational exercise. At T3, statistically significant improvements were noted in the intervention group’s dietary management but not in exercise management. A possible explanation could be that pregnant women may experience obvious weight gain in the T3 compared with that at prepregnancy. The altered body shape potentially results in physical activity restriction, leading pregnant women to prefer weight management through a healthy diet [46]. Therefore, in the T3, physical activity should be emphasized and health education related to suitable exercise should be reinforced. Previous studies have found smartphone applications and other digital interventions to promote a healthy diet among pregnant women with obesity and overweight [47,48]. However, altering the long-term dietary patterns in this intervention study, which focused on psychological cognition, proved difficult, and this might have been influenced by deeply rooted Chinese concepts (ie, “the better the eating during pregnancy, the better the growth of the fetus”). Furthermore, dietary behavioral modification is influenced by external conditions (ie, the convenience of accessing healthy foods), which is beyond the current intervention. Therefore, the ideal dietary intervention should be considered from multiple perspectives, including some external reinforcing factors.

### Strengths and Limitations

This study has several strengths. First, compared with previous similar studies [48-50], the population in this study not only included women who were overweight or obese before pregnancy but also included women of all BMI categories. Second, the behavioral intervention integrated mobile intervention with 2 classical behavioral theoretical models so that the mobile intervention framework was multidimensional, providing a multipronged approach to gestational weight management.

However, this study has several limitations that warrant consideration. First, participants were recruited from a single hospital in Jiangsu Province, which may not adequately represent the entire target population globally. Multiregional, multicenter RCT should be conducted in the future. Second, the outcomes were self-reported by participants, thus potentially leading to reporting and recall bias. Third, the nonrandom sampling method could result in some selection bias. Fourth, the mean and SD values of psychological and behavioral scales were similar across the groups; therefore, the significance of the findings needs to be further examined, and the results need to be carefully interpreted.

### Conclusions

This study assessed the applicability and effectiveness of a gestational weight mobile health intervention program based on the behavioral model integrated with the PMT-IMB model. This mobile intervention enhanced the psychological cognition among pregnant women in information, perceived vulnerability, and response cost, and improved dietary and exercise management. Further research is necessary to confirm the generalizability, operability, and durability of our findings in the practice of mobile health intervention.

## Appendix

Multimedia Appendix 1 The detailed information for the scales.

Multimedia Appendix 2 The operational definition of gestational weight management behaviors.

Multimedia Appendix 3 The detailed intervention contents based on the PMT-IMB (protection motivation theory and information–motivation–behavior skills) model.

Multimedia Appendix 4 CONSORT eHEALTH checklist (V 1.6.1).

## Abbreviations

CONSORT-EHEALTH: Consolidated Standards of Reporting Trials of Electronic and Mobile Health Applications and Online Telehealth
GWG: gestational weight gain
IMB: information-motivation-behavioral skills
PMT: protective motivation theory
PMT-IMB model: protection motivation theory and information–motivation–behavior skills model
RCT: randomized controlled trial
T1: first trimester
T2: second trimester
T3: third trimester

## Data Availability

The datasets generated or analyzed during this study are not publicly available due to the inclusion of protected personal information but are available from the corresponding author on reasonable request.
