# Supplementary material for: Effects of a Mobile Health Intervention Based on Behavioral Integrated Model on Cognitive and Behavioral Changes in Gestational Weight Management: Randomized Controlled Trial
Source: J Med Internet Res. 2025 Mar 10;27:e55844. doi: 10.2196/55844 (PMC11933755; doi:10.2196/55844)
Supplement: Multimedia Appendix 1 [file jmir_v27i1e55844_app1.docx]

**Appendix 1** **The detailed information for the scales**

| **Dimensions** | **Items** | **Items example** | **Five-point scale** | **Cronbach's Alpha** | **Total Cronbach's Alpha** | **Kaiser-Meyer-Olkin value** | **Bartlett sphericity test** |
| --- | --- | --- | --- | --- | --- | --- | --- |
| **Psychological cognitions** | | | | | | | |
| Information | 5 | I have knowledge about the benefits of pregnancy weight management for maternal and fetal outcomes | 1 = absolutely disagree;  5 = absolutely agree | 0.822 | 0.860 | 0.883 | p < 0.001 |
| Behavioral Skills | 5 | I know how to assess whether my weight gain during pregnancy is reasonable |  | 0.879 |  |  |  |
| Perceived Severity | 5 | Inappropriate weight during pregnancy can cause chronic postpartum disease and affect my health |  | 0.899 |  |  |  |
| Perceived Vulnerability | 5 | I think my GWG is appropriate and there is no need for weight management |  | 0.869 |  |  |  |
| Response Efficacy | 5 | If I manage my weight during pregnancy, my risk of adverse pregnancy outcome will be reduced |  | 0.938 |  |  |  |
| Self-efficacy | 5 | I believe that I can overcome the obstacles and difficulties associated with pregnancy weight management |  | 0.927 |  |  |  |
| Response Cost | 5 | Gestational weight management requires exercise, which brings trouble to me | 1 = absolutely agree;  5 = absolutely disagree | 0.915 |  |  |  |
| **Weight Management Behaviors** | | | | | | | |
| Exercise Management | 9 | I exercise 30 minutes or more a day | 1 = never;  5 = always | 0.818 | 0.844 | 0.868 | p < 0.001 |
| Diet Management | 4 | I will cut down on fried, fatty foods and sugary drinks |  | 0.653 |  |  |  |
| Self-monitoring and Regulation | 3 | I measure my weight once a week and record it |  | 0.676 |  |  |  |
| Management Objectives | 4 | Create your own weekly weight gain plan and check to see if pregnancy weight gain is up to par |  | 0.866 |  |  |  |
