# Supplementary material for: Effects of a Mobile Health Intervention Based on Behavioral Integrated Model on Cognitive and Behavioral Changes in Gestational Weight Management: Randomized Controlled Trial
Source: J Med Internet Res. 2025 Mar 10;27:e55844. doi: 10.2196/55844 (PMC11933755; doi:10.2196/55844)
Supplement: Multimedia Appendix 2 [file jmir_v27i1e55844_app2.docx]

**Appendix 2 The operational definition of gestational weight management behaviors**

| **Dimensions** | **Operational definition** |
| --- | --- |
| Exercise management | It refers to various exercise management behaviors taken by pregnant women to maintain appropriate gestational weight gain, including the time and type of exercise. |
| Dietary management | It refers to a series of dietary management behaviors carried out by pregnant women in order to maintain appropriate gestational weight gain. |
| Self-monitoring and regulation | It means that pregnant women carry out weight monitoring, diet tracking, self-regulation and other related behaviors in order to maintain appropriate gestational weight gain. |
| Management objectives | Pregnant women set their own weight growth, dietary and exercise goals according to the appropriate standards for weight gain. |
