# Supplementary material for: Effects of a Mobile Health Intervention Based on Behavioral Integrated Model on Cognitive and Behavioral Changes in Gestational Weight Management: Randomized Controlled Trial
Source: J Med Internet Res. 2025 Mar 10;27:e55844. doi: 10.2196/55844 (PMC11933755; doi:10.2196/55844)
Supplement: Multimedia Appendix 3 [file jmir_v27i1e55844_app3.docx]

**Appendix 3 The detailed intervention contents based on the PMT-IMB model**

| **Variables** | | **Objectives** | **Contents** | **Forms** | **Time: items** |
| --- | --- | --- | --- | --- | --- |
| **Information** | | Increase awareness of weight gain and gestational weight management | The knowledge of appropriate weight gain and gestational weight management. | Image text  Video | 14 weeks: A guideline to gestational weight management. |
|  |  |  |  |  | 15 weeks, 29 weeks: The range of reasonable GWG. |
|  |  |  |  |  | 18 Weeks: Diet and nutrition principles during Pregnancy (Video). |
|  |  |  |  |  | 19 weeks: Routine knowledge of gestational exercise. |
|  |  |  |  |  | 20 weeks: Precautions for gestational exercise. |
|  |  |  |  |  | 21 weeks: Dietary management at all stages of pregnancy. |
|  |  |  |  |  | 24 weeks: Dietary guidelines for pregnant women. |
|  |  |  |  |  | 36 weeks: What to eat at third trimester. |
| **Motivation** | Perceived Severity | Improve awareness of the severity of negative health outcomes | 1、The response to the clinical consequences of negative health outcomes, such as pregnancy complications, macrosomia, neonatal asphyxia, etc.  2、The response to the social consequences of negative health outcomes, such as the impact of family relations and life, personal economy, etc. | Image text | 17 and 30 weeks of gestation: Effects of abnormal weight gain during pregnancy on pregnant women, newborns, etc. |
|  |  |  |  | Expert lectures Peer communication | The harm and severity of abnormal GWG. |
|  | Perceived Vulnerability | Improve awareness of perceived vulnerability to negative health outcomes | The judgment of the likelihood of negative health outcomes and awareness of the presence of associated risk factors. Such as pre-pregnancy obesity or overweight, excessive gestational weight gain, and family genetic history (i.e., history of pregnancy complications). | Expert lectures Peer communication | Knowledge of epidemiology, risk factors and susceptible groups of abnormal weight gain during pregnancy. |
|  | Response Efficacy | Improve awareness of the benefits of gestational weight management | The benefits of gestational weight management. | Image text Video | 16 weeks: Benefits and importance of gestational weight management. |
|  | Self-efficacy | Increased awareness of the possibility of self-acceptance of gestational weight management | The ability to evaluate and judge correctly, as well as practical experience or guidance from others. | Image-text  Expert lectures | Appropriate diet and exercise during the second trimester (Expert lectures). |
|  |  |  |  |  | 23 weeks: Appropriate exercise during the second trimester. |
|  |  |  |  |  | 27 weeks: Dietary contraindications during the second trimester. |
|  |  |  |  |  | 32 weeks: Exercise for the third trimester. |
|  |  |  |  |  | 34 weeks: Late trimester nutritional supplements. |
|  | Response Costs | Improve (reduce) awareness of the difficulties and possible costs of gestational weight management | The correct cognition and judgment of time cost, economic cost and overcoming inherent barriers. | Peer communication | Pregnant women are encouraged to express their existing problems in the app communication group after the question and answer session of expert lectures, and find solutions through the promotion and incentive effect between peers and expert suggestions. In this process, it should pay attention to the psychological changes of pregnant women and enhance their confidence. |
| **Behavioral Skills** | | Improve the behavioral skills needed for gestational weight management | The reasonable diet, scientific exercise, self-monitoring and other skills. | Image text  Weight record | 22 weeks: How to control weight gain at second trimester. |
|  |  |  |  |  | 25 weeks: Weight monitoring and weight assessment in the second and third trimesters. |
|  |  |  |  |  | 26 weeks: The amount of exercise a pregnant woman needs per week. |
|  |  |  |  |  | 28 weeks: Calculation of dietary caloric cards at second trimester. |
|  |  |  |  |  | 31 weeks: Control weight gain at second trimester |
|  |  |  |  |  | 33 weeks: Exercise precautions. |
|  |  |  |  |  | 35 weeks of gestation: Calculation of dietary caloric cards at third trimester. |
|  |  |  |  |  | 37 weeks: Postpartum weight management, postpartum exercise. |
|  |  |  |  |  | Continuous self-recording of weight in the module of weight records. |
